# Circulating microRNAs correlated with the level of coronary artery calcification in symptomatic patients

**DOI:** 10.1038/srep16099

**Published:** 2015-11-05

**Authors:** Wei Liu, Shukuan Ling, Weijia Sun, Tong Liu, Yuheng Li, Guohui Zhong, Dingsheng Zhao, Pengfei Zhang, Jinping Song, Xiaoyan Jin, Zi Xu, Hailin Song, Qi Li, Shujuan Liu, Meng Chai, Qinyi Dai, Yi He, Zhanming Fan, Yu Jie Zhou, Yingxian Li

**Affiliations:** 1 Department of Cardiology, Beijing An Zhen Hospital, Capital Medical University, Beijing, China; 2 State Key Laboratory of Space Medicine Fundamentals and Application, China Astronaut Research and Training Center, Beijing, China; 3 Department of Radiology, Beijing An Zhen Hospital, Capital Medical University, Beijing, China

## Abstract

The purpose of this study was to find the circulating microRNAs (miRNAs) co-related with the severity of coronary artery calcification (CAC), and testify whether the selected miRNAs could reflect the obstructive coronary artery disease in symptomatic patients. Patients with chest pain and moderated risk for coronary artery disease (CAD) were characterized with coronary artery calcium score (CACS) from cardiac computed tomography (CT). We analyzed plasma miRNA levels of clinical matched 11 CAC (CACS > 100) and 6 non-CAC (CACS = 0) subjects by microarray profile. Microarray analysis identified 34 differentially expressed miRNAs between CAC and non CAC groups. Eight miRNAs (miR-223, miR-3135b, miR-133a-3p, miR-2861, miR-134, miR-191-3p, miR-3679-5p, miR-1229 in CAC patients) were significantly increased in CAC plasma in an independent clinical matched cohort. Four miRNAs (miR-2861, 134, 1229 and 3135b) were correlated with the degree of CAC. Validation test in angiographic cohort showed that miR-134, miR-3135b and miR-2861 were significantly changed in patients with obstructive CAD . We identified three significantly upregulated circulating miRNAs (miR-134, miR-3135b and 2861) correlated with CAC while detected obstructive coronary disease in symptomatic patients.

Most individuals aged over 60 years have progressively enlarging deposits of calcium mineral in their major arteries^1^, which in the coronary vasculature weaken vasomotor responses and alter atherosclerotic plaque stability^2^. Coronary artery calcification (CAC), as a sign of atherosclerosis, reflects the long-term impact of elevated coronary artery disease (CAD) risk and independently predicts future risk of CAD events in symptomatic patients^3^,^4^,^5^. In the symptomatic patient, CAC score measure from electron-beam computed tomography (EBCT) and multi-detector computed tomography (MDCT) can also be used as a noninvasive diagnostic technique for detecting obstructive CAD.

Vascular calcification as a cell-regulated process characterized by osteogenic transition of vascular smooth muscle cells (SMCs), valvular interstitial cells (VIC) or stem cells^6^,^7^. Recent advances have identified microRNAs (miRNAs), which are noncoding, ~22-nucleotide-long RNAs that function as post-transcriptional regulators of gene expression, as key regulators of CAC by directing the complex genetic reprogramming of SMCs and the functional responses of other cell types relevant for vascular calcification. MiRNAs have also attracted interest as putative circulating biomarkers of cardiovascular and other diseases because of their stability in plasma and serum^8^, and differential profiles of circulating miRNAs have been reported for cardiac hypertrophy^9^, acute myocardial infarction (AMI)^10^,^11^,^12^, heart failure^13^, coronary artery disease^14^, and diabetes mellitus (DM)^15^. However, signatures of circulating miRNAs have not been characterized in individuals with CAC, which is the subject of this study.

## Materials and Methods

### Patient population

Two cohorts of patients were included into this study. The first cohort consists of patients aged 40–65 years with moderate probability of coronary artery disease (CAD) recruited from the Cardiology outpatient clinic at Beijing An Zhen Hospital. The second cohort consists of symptomatic patients referred to our institution for coronary angiography. For either of these two cohorts, those with known chronic renal failure, insulin dependent DM, previous history of AMI, percutaneous coronary artery intervention or coronary artery bypass graft, or with degenerative aortic valve disease or acute coronary syndrome were excluded from the study. All selected patients provided informed consent, and the study protocol conformed to the ethical guidelines of the 1975 Declaration of Helsinki. The study was approved by the institutional review board of Beijing An Zhen Hospital and the Institutional Review Board of the China Astronaut Research and Training Center.

### Study Flow

**Figure 1:**
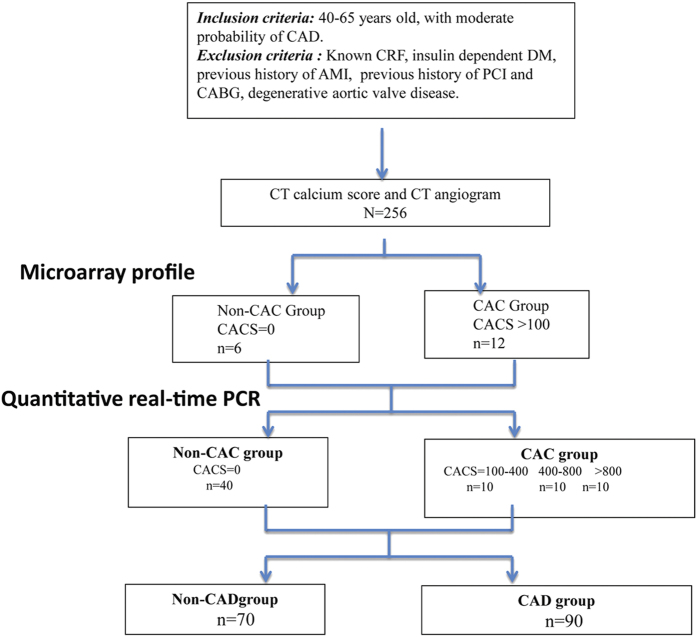
Study flow chart. CRF, chronic renal failure; DM, diabetes mellitus; AMI, acute myocardial infarction; PCI, percutaneous coronary intervention; CABG, coronary artery bypass graft; CT, computed tomography; CAC, coronary artery calcification; PCR, polymerase chain reaction; CACS, coronary artery calcium score.

The study flow is shown in Fig. 1. All selected study participants in first cohort (CTA cohort) had coronary artery calcium score determined by coronary CT angiogram unless latter was not possible (allergy to intravenous contrast, unable to hold breath, and/or arrhythmia). Blood samples were collected and stored per protocol for further testing (see below). Clinically matched patients (age, gender, history of DM) from the non-CAC group (CACS = 0, *n* = 6) and CAC group (CACS > 100, *n* = 12) were selected for microarray profile study, and differentially expressed miRNAs identified were further tested in another clinically matched cohort which included 40 patients in the non or low CAC group (CACS = 0, *n* = 40) and 30 patients in the CAC group (CACS 100–400, *n* = 10; CACS 400–800, *n* = 10; CACS > 800, *n* = 10). A separated cohort consisted 160 patients undergoing coronary angiography (Patients with obstructive CAD n = 90, and patients without CAD n = 70 as controlled group). All patients had blood test for miRNA as mentioned below.

### CACS and coronary CT angiogram

CT coronary artery angiogram and CACS were performed for patients with moderate probability of CAD. All patients were in normal sinus rhythm capable of a breathhold sufficient for CTA. Coronary calcium score was obtained using imaging obtained with a second-generation dual source computed tomography (Somatom Definition Flash, Siemens Healthcare, Forchheim, Germany) with the adaptive prospective CorAdSeq model. The imaging parameters were as follows: detector collimation, 2 × 64 × 0.6 mm; slice acquisition, 2 × 128 × 0.6 mm by means of a z-flying focal spot; and gantry rotation time, 280 msec. The tube voltage was adjusted according to each patient’s body mass index (BMI): 100 or 120 kV for BMI < or ≥25.0 kg/m^2^, respectively. Automatic exposure control system-based tube current modulation (Care-DOSE; Siemens Medical Solutions) was used in all patients with two-data acquisition techniques. For image acquisition, patients were placed in the supine position. After a 65–75 ml bolus of intravenous contrast (Isovue 370; Bracco Diagnostics, Princeton, NJ) followed by a 40 ml saline flush both at a rate of 3.8–4.5 ml/s, image acquisition was performed starting 5 mm above the take-off of the left main coronary artery to determine optimal time for CTA performance. All helical scan data were obtained with prospective electrocardiographic (ECG) gating. All ECG-gated data sets were reconstructed at 70%–80% of the R-R interval for the identification of mid-systole. Additional reconstruction was performed at 40%–50% of the cardiac cycle to define end-systole and beginning-diastole. Image reconstruction was performed immediately after scanning to assure motion-free arterial scans. A standard calcium scoring kernel (B35f) was used for reconstruction of the CT data. Calcium (Ca)-scoring was performed on the reconstructed image sets with commercially available software (Syngo CaScore, Siemens, Forchheim, Germany). Agatston scoring, described in detail elsewhere^16^ was used for calcium score. A standard scoring threshold of 130 HU was used during the procedure. The Ca-score of the total artery was used instead of the Ca-scores of individual calcifications.

For coronary artery stenosis analysis, optimal phase reconstruction was assessed by comparing differing phases of the coronary cycle to identify images with minimal arterial motion, which were then highlighted for analysis. The CTA was evaluated with maximum intensity projections in cardiocentric views for optimal viewing of individual coronary artery segments. Three-dimensional volume-rendering and curved multiplanar reformation techniques were also used to enhance detection of obstructive CAD. A stenosis of >50% was considered indicative of a significant lesion^16^.

### Coronary angiography

Coronary angiography was performed in a separated cohort according to Judkin’s technique with a minimum of 4 views of the left system and 2 views of the right system. The maximum percent diameter stenosis in any coronary segment was visually assessed by at least two experienced interventionists. Narrowing of the lumen diameter by 50% was defined as significant CAD.

### Blood collection

Peripheral blood samples (2 ml) were collected into EDTA-containing tubes. Whole blood was centrifuged at 1200 g for 15 min at room temperature within 30 min after blood collection, and the supernatant fluid was transferred into microcentrifuge tubes, followed by a second centrifugation at 12, 000 g for 10 min at 4 °C to remove cellular debris. Plasma was then aliquoted and stored at −80 °C until use.

Total RNA was extracted from 400 μl of plasma using the mirVanaTM RNA Isolation Kit (Applied Biosystems, Foster City, CA, USA) according to the manufacturer’s specifications, and eluted with 100 μl of nuclease free water. Subsequently, we concentrated the RNA in a final volume of 20 μl. The yield of RNA was determined using a NanoDrop ND-1000 spectrophotometer (Nanodrop Technologies, Wilmington, DE, USA).

### MicroRNA microarray expression profiling

Total RNAs from plasma from 12 and 6 individuals with and without CAC, respectively, were used for microRNA microarray profiling. Total RNA (100 ng) was quantified by the NanoDrop ND-2000 (Thermo Scientific), and RNA integrity was assessed using Agilent Bioanalyzer 2100 (Agilent Technologies). Sample labeling, microarray hybridization and washing were performed as per the manufacturer’s standard protocols. Briefly, total RNA were dephosphorylated, denatured and then labeled with Cyanine-3-CTP. After purification the labeled RNAs were hybridized onto the microarray (Release 19.0, Agilent) containing probes for 2006 human microRNAs. After washing, the arrays were scanned with the Agilent Scanner G2505C (Agilent Technologies) and the scanned images were analyzed using Agilent Feature Extraction Software (Agilent Technologies).

### Circulating miRNA extraction and detection by Quantitative RT-PCR

miRNA from 200 μl plasma was extracted using the miRNeasy Serum/Plasma Kit, according to the manufacturer’s recommendations (Qiagen, Germany). Briefly, samples were supplemented (after addition of QIAzol) with 3.5 μl miRNeasy Serum/Plasma Spike-In Control (1.6 × 10^8^ copies/μl working solution) (Qiagen, Germany). We demonstrated that the cel-miR-39 could be used for normalization of the RNA preparation. The amount and purity of RNA was estimated by quawell micro volume spectrophotometer (Quawell, USA). Subsequently, miRNA was transcribed to cDNA using the miScript II RT Kit (Qiagen, Germany). Diluted cDNA (1:10) were used for detecting miRNA expression by Q-PCR using the miScript SYBR Green PCR Kit with miScript Primer Assay (Qiagen, Germany). The relative expression level of miRNA was determined by the cycle number via Q-PCR, with levels normalized to the average of cel-miR-39 using the 2-ΔΔCT method.

### Statistical analyses

Data are expressed as mean + standard deviation (SD) or proportions where appropriate. Two-tail student’s t-test was used to compare clinical characteristics between groups where appropriate. MicroRNA data are presented as fold change relative to cel-miR-39 expression in each sample. Receiver operating characteristics (ROC) curves and the area under the ROC curve (AUC) were established to evaluate the diagnostic value of plasma microRNAs whose levels differed between individuals with and without CAC. An AUC of 0.5 indicates classifications assigned by chance. Based on ROC analysis, the best statistical cutoff values of plasma microRNAs were calculated, and the sensitivity and specificity for selected cutoff points were then assessed. All statistical analysis was performed using Graphpad Prism 5.01 for Windows (Graphpad Software Inc., San Diego, CA, USA). Differences were considered statistically significant at a value of *P* < 0.05 (two-tailed).

## Results

### Patient characteristics

The microarray cohort of subjects included 12 individuals with CAC (one RNA sample failed to pass the quality control test) and 6 non-CAC controls. For independent validation, in addition to the microarray cohort, we studied a second group composed of 30 individuals with CAC (CAC > 100) and 40 non-CAC controls (CACS = 0). Their characteristics are summarized in Table 1.

### Expression profiles of microRNAs in the plasma of individuals with CAC

**Figure 2:**
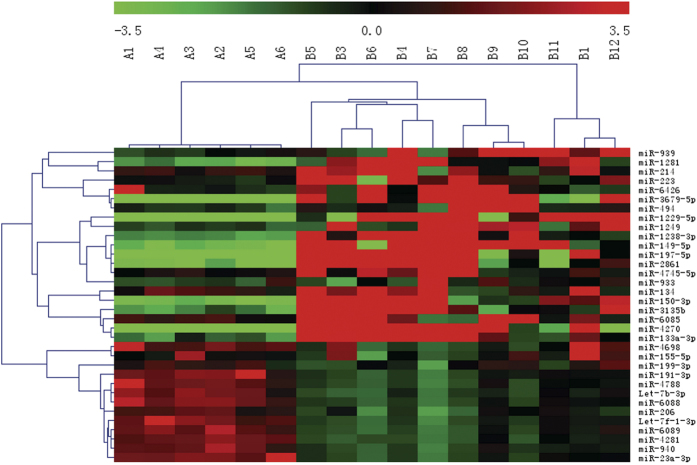
Heat map of microRNA (miRNA) microarray expression data from plasma samples of individuals with (n = 11) and without (n = 6) coronary artery calcification. miRNA expression is hierarchically clustered on the y axis, and plasma samples from individuals with and without coronary artery calcification are hierarchically clustered on the x axis. The legend on the right indicates the miRNA represented in the corresponding row. The relative miRNA expression is depicted according to the color scale shown on the right. Red indicates upregulation; and green, downregulation. Numbers with A indicate non-coronary artery calcification samples; numbers with B, coronary artery calcification sample.

To determine the differential miRNA levels in individuals with CAC, we comparatively profiled plasma miRNA expression of 11 and 6 individuals with and without CAC, respectively. The levels of circulating miRNAs significantly differed between CAC and non-CAC groups, as illustrated in the heat map shown in Fig. 2. Of 2006 miRNAs detected on the microarray, 34 miRNAs were found to be differentially expressed in individuals with CAC relative to those without CAC (*P* < 0.05). Levels of 21 miRNAs were increased and those of 13 miRNAs were decreased in the CAC vs. non-CAC groups. Fold changes in levels of miRNAs in the array are shown in Table 2.

### Quantitative reverse transcription polymerase chain reaction validation of the profiling data

**Figure 3:**
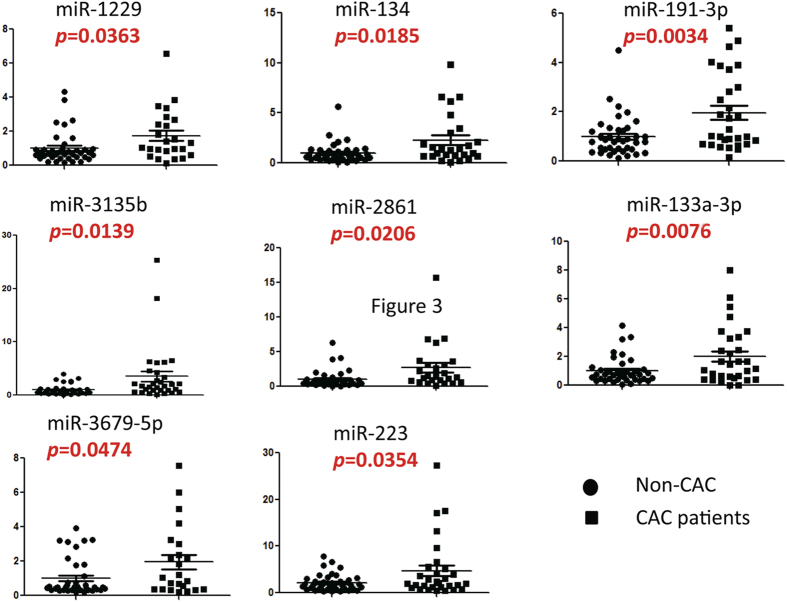
Validation of microRNA (miRNA) microarray data by quantitative reverse-transcription polymerase chain reaction. The microarray cohort included 30 individuals with coronary artery calcification (CAC) and 40 controls without coronary artery calcification (non-CAC). The relative levels of miRNAs were normalized to levels of the control (cel-miR-39). The P values were calculated by 2-tailed Student t test.

To confirm miRNA profiling findings, we measured the levels of the 34 dysregulated miRNAs on the basis of their fold changes and P values in an independent cohort (40 non-CAC and 30 CAC individuals) via quantitative RT-PCR. The relative level of each miRNA in CAC vs. non-CAC individuals is shown in Fig. 3. Consistent with the profiling data, the levels of 8 miRNAs were increased (*P* < 0.05) in individuals with CAC compared to non-CAC controls (Fig. 3). Our results demonstrated fold increases of 3.52 for miR-3135b (*P* < 0.05); 2.85 for miR-133a-3p (*P* < 0.01); 2.63 for miR-2861 (P < 0.05); 2.28 for miR-134 (*P* < 0.05); 2.23 for miR-223 (*P* < 0.05); 1.96 for miR-191-3p (*P* < 0.01); 1.85 for miR-3679-5p (*P* < 0.05); and 1.74 for miR-1229 (*P* < 0.05) in individuals with CAC. Difference between CAC and non-CAC groups was not significant for levels of other miRNAs, and there were no significantly downregulated miRNAs in individuals with CAC.

### ROC analysis for differential expression of circulating miRNAs

**Figure 4:**
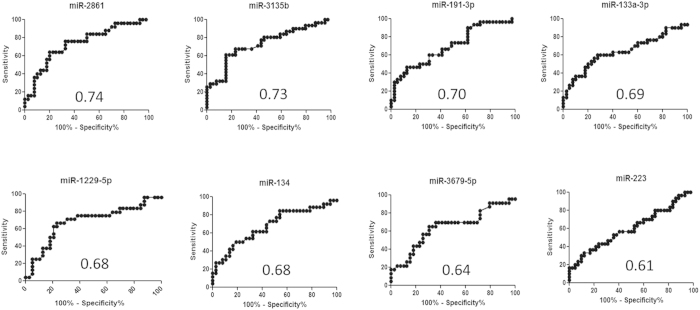
AUC analysis of receiver-operating characteristics. The area under the curve (AUC) (values given on the graphs) for miRNAs with significantly increased circulating levels was calculated for the coronary artery calcification group.

The findings of 8 upregulated circulating miRNAs in individuals with CAC indicate that circulating miRNA levels allow to distinguish CAC from coronary artery disease patients. To assess the potential diagnostic value of miRNAs with significantly changed levels, a ROC curve analysis was performed. The associated AUC was used to confirm the diagnostic value for each miRNA (Fig. 4). As shown in Table 3, 6 of the 8 significantly increased miRNAs had an optimal area value under the curve (AUC > 0.65) . As shown in Fig. 4, the AUC of miR-2861 was the highest, reaching 0.7400 ([95% confidence interval = [0.61–0.87], *P* < 0.001). The AUC of the remaining five miRNAs was 0.73 for miR-3135b ([0.61–0.86], *P* < 0.001); 0.70 for miR-191-3p ([0.58–0.83], *P* = 0.0046); 0.69 for miR-133a-3p ([0.55–0.82], *P* = 0.0096); 0.68 for miR-1229-5p ([0.54–0.83], *P* = 0.015); and 0.68 for miR-134 ([0.54–0.82], *P* = 0.015). The sensitivity and specificity associated with the optimal cutoff points are also shown in Table 3. MiR-2861 showed the highest sensitivity of 76.0% and a specificity of 67.5%.

### Correlation of circulating miRNA level with coronary artery calcium score

**Figure 5:**
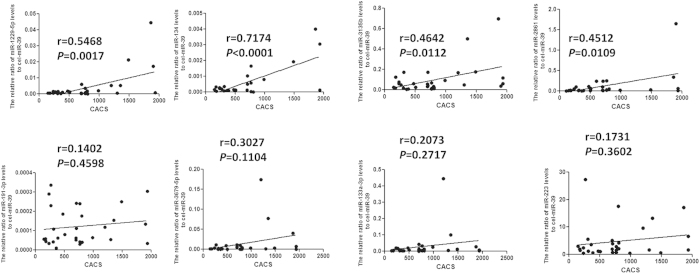
Pearson Correlation Coefficients between significantly increased miRNAs and coronary artery calcium score.

After determining the differential miRNA expression pattern, we examined correlation of these 8 miRNAs with the level of CACS. The levels of miR-1229-5p, miR-134, miR-2861 and miR-3135bp, but not those of miR-191-3p, miR-133a-3p, miR-3679-5pb and miR-223 showed significant positive correlation with CACS (Fig. 5). Consistent with the results of ROC analysis, the levels of circulating miR-1229, miR-134, miR-3135b and miR-2861 were significantly associated with CACS. MiR-134 showed the highest positive correlation with CACS (r = 0.7174, *P* < 0.0001).

### Independent Validation of microRNA Expression in patients undergoing coronary angiography

**Figure 6:**
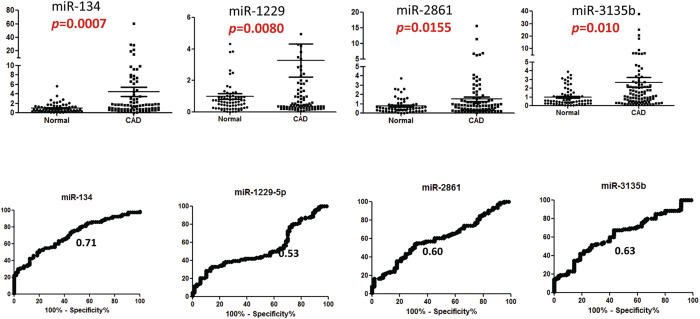
The levels of circulating miR-1229-5p, miR-134, miR-3135b and miR-2861 in patients with CAD and healthy control subjects. Non CAD group, n = 70; CAD group, n = 90. The P values were calculated by 2-tailed Student t test.

The selected circulating miR-1229, miR-134, miR-3135b and miR-2861 were further tested in 160 patients (90 CAD patients and 70 age, sex, DM matched control subjects.) Undergoing coronary angiography to validate the potential function of these makers in detecting obstructive coronary artery disease. As shown in figure 6 and Table 4, the levels of all these 4 miRNAs in CAD patients were all significantly different from those of controls (miR-134, *p* = 0.0007; miR-1229, *p* = 0.008; miR-3135b, *p* = .01; miR-2861, *p* = 0.015). ROC analysis showed that AUC was highest for miR-314 (0.71, *p* < 0.0001,), then miR-3135b (0.63, *p* = 0.038) and miR-2861 (0.6, *p* = 0.03), but no significant for miR-1229-5p (0.53, *p* = 0.53). miR-134 showed the highest sensitivity of 72.5% and a specificity of 80.0%.

## Discussion

In this study, we investigated the association of certain plasma circulating miRNAs with coronary calcification and their clinical significance. We characterize the circulating miRNA profile in CAC patients and identify circulating miRNAs which can best reflect the level of CAC in symptomatic patients with moderate risk of CAD and further tested whether those miRNA can detect the presence of obstructive CAD. Expression profiling of circulating miRNA revealed significant upregulation of 23 miRNAs and down regulation of 11 miRNAs, while only 8 of the upregulated miRNAs were further validated in a larger patient cohort using qRT-PCR. We found that 6 of these 8 miRNAs had an ROC curve that distinguished the CAC patients. Importantly, significant positive correlations were identified between CACS and four miRNAs (miR-1229-5p, miR-134, miR-3135b and miR-2861), eventually 3 miRNAs (miR-134, miR-3135b and miR-2861) showed clinical significance in detecting the presence of obstructive CAD in symptomatic patients.

Some of differentially expressed circulating miRNAs appear to be involved in the biologic processes of CAC from previous *in vivo* study. miR-2861 plays a positive regulatory role in osteoblast differentiation; it targets histone deacetylase 5 (HDAC5) which is an enhancer of runt-related transcription factor2 (Runx2) degradation. *In vivo*, silencing of miR-2861 in mice using antagomir represses Runx2 protein expression and inhibits bone formation. Also, mutation of pre-miR-2861 in patients can cause primary osteoporosis^17^,^18^. Because the key feature in vascular calcification is osteogenic transition of SMCs, miR-2861 might function as an enhancer of osteogenic differentiation of SMCs. Our investigation in plasma showed a strong upregulation of miR-2861 in CAC patients. The increased circulating miR-2861 level may reflect CAC progression.

The human miR-134 is one of the most extensively present in plasma. Some previous studies used miR-134 as biomarker in other diseases^19^,^20^, such as lung adenocarcinoma-associated malignant pleural effusion^21^ and acute myocardial infarction^12^. miR-134 also acted as metastasis suppressor in carcinoma by targeting integrin β1^22^, which plays a positive regulatory role in osteoblast differentiation^23^,^24^. Our investigation in plasma showed a strong upregulation of miR-134 in CAC patients. However, cardiovascular calcification is frequently accompanied by decreased bone mineral density or osteoporosis in human and animal models, which is called the “calcification paradox”^25^,^26^. The upregulation of circulating miR-134 in CAC patients may reflect another type of “calcification paradox”.

To our knowledge, the function of miR-1229 and miR-3135b remains unknown, and only one report documented significantly higher serum exosomal level of miR-1229 in a primary colorectal cancer patient suggesting that it might be a promising biomarker for non-invasive diagnosis of the disease^27^. Functional analysis in targetScan 6.0 showed that miR-1229 and miR-3135b could target different genes involved in vascular development and in basic biological processes such as cell-cell signaling, DNA replication, cell death and survival.

MiRNAs have been demonstrated to play crucial roles in many physiological and pathophysiological processes^28^. miRNAs contribute to different forms of CVD, and the changes in circulating miRNAs can be detected as a consequence of pathological changes^10^,^29^. Circulating miRNAs have been identified as biomarkers for various physiological and pathological conditions^10^,^30^. The microarray chip for miRNA provides a powerful approach for global circulating miRNA characterization, and it is simple to universally perform quantitative validation using real time-PCR^29^. It has been suggested that the discovery-validation procedure for circulating miRNA biomarkers will be more efficient than that for traditional proteomic biomarker identification.

The study provided significant clinical significance, To our knowledge, this is the first study to evaluate the circulating miRNA fingerprint in CAC patients which was traditionally evaluated by CAC score, CTA and other imaging technique^3^,^31^.

Calcification of the coronary arteries is highly correlated with atherosclerosis^32^. Early detection of CAC is important for identifying subclinical atherosclerosis and predict the risk of CAD^3^,^33^ . By identifying specific circulating miRNAs for CAC, we are providing a novel way of identifying the severity of CAC which can also function as potential biomarkers for the presence of obstructive CAD. Moreover the results of the current study highlight that further insight into the function of circulating miRNAs in the process and progression of CAC.

### Limitation

Even though the coronary calcification has significant meaning in predicting the future risk of CAD, however, its role in plaque instability is still debatable. Lesions associated with unstable angina or infarction tend to have multiple, small calcium deposits, in “spotty” or “speckled” patterns, whereas those instable angina are associated with few, large calcium deposits. Our study included symptomatic patents, and did not evaluate the plaque stability by OCT, IVUS or clinical events. Future studies could be performed to identify certain miRNAs that contribute to the instability of calcified lesions and associated with worse future clinical outcomes.

In conclusion, the present study provides the first evidence of an altered circulating miRNA expression profile in adult CAC patients. Three circulating miRNAs (miR-134, miR-3135b and miR-2861) correlated with CACS and associated with the presence of obstructive CAD.

## Additional Information

**How to cite this article**: Liu, W. *et al.* Circulating microRNAs correlated with the level of coronary artery calcification in symptomatic patients. *Sci. Rep.* **5**, 16099; doi: 10.1038/srep16099 (2015).

## Figures and Tables

**Table 1:** Clinical characteristics of individuals with coronary artery calcification.

	Non-CAC(n = 40)	CAC (n = 30)	*P* value
Male gender	25(62.5%)	23(76.7%)	0.315
Age	59.6 ± 8.2	59.6 ± 6.2	0.995
DM(mmol/L)	5(12.5%)	7(23.4%)	0.23
Hypertension(mmHg)	17(42.5%)	17(56.7%)	0.241
Smoker	12(30.0%)	10(33.3%)	0.766
LDL(mmol/L)	2.9 ± 0.7	2.4 ± 0.7	0.021
TG(mmol/L)	1.5 ± 1.2	1.8 ± 1.1	0.201
CHO(mmol/L)	4.6 ± 1.3	4.3 ± 0.9	0.33
HDL(mmol/L)	1.5 ± 0.9	1.2 ± 0.2	0.05
ALP(U/L)	91.7 ± 40.8	95.5 ± 44.7	0.819
BMI(kg/m^2^)	25.3 ± 3.6	25.1 ± 3.3	0.84
CACS	11.3 ± 24.1	730.4 ± 524.1	<0.0001
CAD	8(20%)	24(80%)	<0.0001

DM:Diabetes mellitus; LDL: Low density lipoprotein; HDL: High density lipoprotein; TG:Triglyceride; CHO: Cholesterol; ALP: Alkaline phosphatase; BMI: Body mass index; CACS: Coronary Artery Calcification score. CAD coronary artery disease (coronary narrowing more than 50% in at least one major vessel from coronary CTA).

**Table 2:** Properties of microRNAs Differentially Expressed in coronary artery calcification Patients Compared With non-calcification Controls.

Upregulated miRNAs	Fold change	*P*value	Downregulated miRNAs	Fold change	*P*value
miR-1229-5p	6.36	<0.001	Let-7b-3p	0.38	<0.01
miR-1238-3p	18.23	<0.05	Let-7f-1-3p	0.11	<0.05
miR-1249	5.64	<0.01	miR-191-3p	0.29	<0.05
miR-1281	11.58	<0.05	miR-206	0.49	<0.05
miR-133a-3p	7.69	<0.05	miR-23a-3p	0.04	<0.01
miR-134-5p	4.9	<0.05	miR-4281	0.31	<0.001
miR-149-5p	8.12	<0.05	miR-4698	0.84	<0.05
miR-150-3p	4.45	<0.05	miR-4788	0.15	<0.05
miR-155-5p	2.23	<0.05	miR-6088	0.17	<0.001
miR-197-5p	9.36	<0.05	miR-6089	0.32	<0.001
miR-199-3p	1.89	<0.05	miR-940	0.47	<0.01
miR-214	8.32	<0.05			
miR-223	6.39	<0.05			
miR-2861	4.18	<0.05			
miR-3135b	11.84	<0.05			
miR-3679-5p	9.42	<0.05			
miR-4270	5.86	<0.05			
miR-4745-5p	9.27	<0.05			
miR-494	9.82	<0.05			
miR-6085	10.18	<0.05			
miR-6426	6.77	<0.05			
miR-933	3.48	<0.01			
miR-939	9.42	<0.05			

**Table 3:** Sensitivity and specificity of the regulated miRNAs in the plasma of CAC and non-CAC individuals.

miRNAs	AUC	95%CI	*P* value	Sensitivity	Specificity
miR-2861	0.74	0.61–0.87	<0.001	76.0%	67.5%
miR-3135b	0.73	0.61–0.86	<0.001	61.3%	84.6%
miR-191-3p	0.70	0.58–0.83	0.0046	46.7%	84.6%
miR-133a-3p	0.69	0.55–0.82	0.0096	64.3%	72.5%
miR-1229-5p	0.68	0.54–0.83	0.015	66.7%	76.9%
miR-134	0.68	0.54–0.82	0.015	50.0%	81.1%
miR-3679-5p	0.64	0.49–0.79	0.06	65.2%	69.2%
miR-223	0.61	0.47–0.74	0.13	33.3%	88.1%

Data were evaluated via a cut-off point.

**Table 4:** Sensitivity and specificity of the regulated miRNAs in the plasma of CAD and non-CAD individuals.

miRNAs	AUC	95%CI	*P* value	Sensitivity	Specificity
miR-134	0.71	0.63–0.79	<0.0001	72.5%	80.0%
miR-3135b	0.63	0.55–0.72	0.0038	51.6%	72.9%
miR-2861	0.60	0.51–0.69	0.031	54.2%	69.4%
miR-1229-5p	0.53	0.44–0.62	0.47	31.8%	87.1%

Data were evaluated via a cut-off point.
